# Supplementary material for: The Effect of Guisangyou Tea on Abnormal Lipid Metabolism in Mice Induced by High-Fat Diet
Source: Foods. 2023 May 27;12(11):2171. doi: 10.3390/foods12112171 (PMC10252605; doi:10.3390/foods12112171)
Supplement: Supplementary file 1 [file foods-12-02171-s001.zip › foods-2389384-supplementary.pdf]

foods

Article

The Effect of Guisangyou Tea on Abnormal Lipid Metabolism in Mice Induced by High-Fat Diet

Yan Zhu [1,†], Xianghui Zhou [2,†], Nan Ling [2], Qiming Yu [2,*], Huijuan Wang [1] and Qizhen Du [1,*]

[1] College of Food and Health Sciences, Zhejiang A & F University, Hangzhou 311300, China; yueliangyan@163.com (Y.Z.); wanghuijuan1016@126.com (H.W.)
[2] College of Public Health, Guilin Medical University, Guilin 541100, China; ZHOUXH_0472@163.com (X.Z.); lingnan0118@163.com (N.L.)
* Correspondence: qm_yu19@glmc.edu.cn (Q.Y.); qizhendu@163.com (Q.D.)
† These authors contributed equally to this work.

Supplementary Materials

Table S1. The nutrient composition of normal diet and high-fat diet.

Ingredient	Normal diet (g)	High-fat diet (g)
Corn starch	397.5	227.5
Casein	200	200
Dyetrose	132	132
Lard	0	170
Sucrose	100	100
Soybean oil	70	70
α-cellulose	50	50
Minerals mixture [a]	35	35
Vitamins mixture [a]	10	10
DL-Methionine	3	3
Choline bitartrate	2.5	2.5
Total weight	1000	1000

[a] Minerals and Vitamins mixture according to AIN-93 diet (Teklad, Madison, WI, USA)

Table S2. The mouse organ indexes of the control group (CON), high fat diet group (HFD) and high fat diet-WE treatment group (WE)

Group	Organ index/%			
	Liver	Kidney	Spleen	Heart
CON	3.913 ± 0.143^b	1.223 ± 0.054^a	0.341 ± 0.033^a	0.461 ± 0.025^a
HFD	4.928 ± 0.135^a	1.226 ± 0.128^a	0.322 ± 0.025^a	0.440 ± 0.025^a
WE	4.379 ± 0.164^b	1.218 ± 0.138^a	0.352 ± 0.034^a	0.454 ± 0.024^a

Significant differences between groups are denoted by different letters ($P < 0.05$).
